# The Real-World Effectiveness of Inactivated COVID-19 Vaccines in Zimbabwe During the Omicron Variant Dominance: A Test-Negative Case–Control Study

**DOI:** 10.3390/vaccines12121303

**Published:** 2024-11-22

**Authors:** Azure Tariro Makadzange, Patricia Gundidza, Kimberly Cheryl Chido Konono, Margaret Gurumani, Chiratidzo Ndhlovu

**Affiliations:** 1 Mutala Trust, Harare, Zimbabwe; patricia.gundidza@crmgresearch.com (P.G.); margaret.gurumani@crmgresearch.com (M.G.);; 2 Department of Medicine, University of Zimbabwe College of Health Sciences, Harare, Zimbabwe

**Keywords:** COVID-19, vaccine effectiveness, inactivated vaccines, SARS-CoV-2, omicron variant, test-negative case–control study, Zimbabwe, BBIP-CorV, Coronovac, Sinopharm, Sinovac

## Abstract

Background/Objectives: The COVID-19 pandemic has significantly impacted global health, with varying vaccine effectiveness (VE) across different regions and vaccine platforms. In Africa, where vaccination rates are relatively low, inactivated vaccines like BBIP-CorV (Sinopharm) and Coronovac (Sinovac) have been widely used. This study evaluated the real-world effectiveness of licensed inactivated COVID-19 vaccines in Zimbabwe during a period dominated by Omicron variants. Methods: We conducted a prospective, test-negative, case–control study among symptomatic adults across six Zimbabwean provinces from November 2022 to October 2023. Participants were categorized based on vaccination status, and nasopharyngeal swabs were collected for SARS-CoV-2 PCR testing. Vaccine effectiveness was assessed using conditional logistic regression, adjusting for various covariates such as age, sex, and comorbidities. Results: Among 5175 participants, 701 tested positive for SARS-CoV-2 and 4474 tested negative. The overall adjusted VE against symptomatic COVID-19 was 31% (95% CI: 5.3–49.7%) among verified vaccinated individuals. Boosted individuals demonstrated a higher VE of 59.8% (95% CI: 40.3–72.9%). VE decreased significantly to 24% (95% CI: −4.1–44.8%) in individuals vaccinated over a year prior. Similar VE was observed for BBIP-CorV (36.8%, 95% CI: 11.4–54.9%) and Coronovac (38.1%, 95% CI: 16.3–54.2%). Conclusions: This study indicates modest protection from inactivated COVID-19 vaccines against symptomatic Omicron infection, with significant enhancement following booster doses. These findings highlight the need for continued vaccine evaluation, particularly in resource-limited settings, to inform public health strategies and optimize vaccination programs.

## 1. Introduction

The COVID-19 pandemic, caused by severe acute respiratory syndrome coronavirus 2 (SARS-CoV-2), has emerged as one of the most devastating global health crises in recent history [1]. Since the first reported case in Wuhan, China, in December 2019, the virus has spread rapidly worldwide, resulting in over 760 million cases and 6.9 million deaths [2]. The RNA virus has replicated repeatedly, leading to the emergence of new variants with genomic mutations associated with alterations in severity, transmissibility, and immune escape [3].

The global response to the pandemic has been unprecedented, with researchers and pharmaceutical companies worldwide driving innovative efforts to develop effective vaccines and respond to emerging variants. In Africa, where COVID-19 vaccination rates have been among the lowest globally, seven types of COVID-19 vaccines have been introduced, including inactivated vaccines such as Sinopharm (China National Pharmaceutical Group Corporation, Beijing, China) (BBIBP-CorV) and Sinovac (Sinovac Biotech, Beijing, China) (Coronovac) [4].

Clinical trials and real-world studies have demonstrated promising results for these inactivated vaccines. Phase III data for BBIBP-CorV, following two doses 21 days apart, reported vaccine efficacy of 78.1% (95% CI: 64.8–86.3%) against symptomatic disease [5]. Coronovac’s phase III trial in Brazil showed 50.7% efficacy (95% CI: 36–62%) against symptomatic disease and 100% (95% CI: 17–100%) against severe disease after two doses given 14 days apart [6]. Trials in Indonesia and Turkey demonstrated efficacies of 65% (95% CI: 20–85%) and 83.5% (95% CI: 65.2–92.1%), respectively, against symptomatic disease [7,8].

Vaccine efficacy in Africa has shown considerable variation, ranging from 41% to 100% across different vaccine platforms, including mRNA, non-replicating viral vectors, and protein subunit vaccines [9]. However, studies on the effectiveness of inactivated vaccines in Africa are limited. Vaccine effectiveness (VE) studies with inactivated vaccines in Egypt and Morocco demonstrated VE of 67% (95% CI: 43–80%) and 64% (95% CI: 59–69%) against symptomatic disease several months post-vaccination, respectively [10,11]. Another Moroccan study retrospectively evaluated the real-world effectiveness of BBIBP-CorV, reporting a VE of 88.5% (95% CI: 85.8–90.7%) against severe disease [12]. These studies were either in specialized populations, e.g., healthcare workers; retrospective analyses conducted shortly after vaccines were administered; or within limited geographies, e.g., North Africa, predominantly.

Approximately 20% of vaccines acquired in Africa were inactivated vaccines (6.8% Coronovac and 13.6% BBIBP-CorV), with countries such as Zimbabwe relying almost entirely on inactivated vaccines in their vaccination programs [13]. While clinical trials provide important insights into efficacy, real-world evidence of VE is critical for evaluating effectiveness, particularly in key groups such as pregnant women, people living with HIV, or people with comorbidities that may have been underrepresented in clinical trial cohorts. Additionally, with the continuous emergence of novel variants, monitoring VE and the impact of boosting is significant for assessing the effectiveness of public health measures [14]. In low- and middle-income countries (LMICs) with fragile health systems that lack robust record-keeping or large electronic health records (EHRs), prospective observational studies such as test-negative case–control designs offer a cost-effective and practical approach to assess vaccine effectiveness [15,16]. To provide critical, real-world data, we conducted a test-negative case–control study in Zimbabwe to estimate the effectiveness of licensed inactivated COVID-19 vaccines against laboratory-confirmed symptomatic COVID-19 disease at a time when Omicron variants were dominant [17].

## 2. Materials and Methods

We conducted a prospective test-negative case–control study among symptomatic adults at treatment centers across six Zimbabwean provinces from November 2022 to October 2023 to estimate the vaccine effectiveness of licensed inactivated COVID-19 vaccines against laboratory-confirmed COVID-19 disease. Eligible participants were adults (≥18 years) presenting at a health facility within 10 days of symptom onset and meeting the WHO surveillance case definition for COVID-19 [18]. According to WHO, a suspected case of SAR-CoV-2 will either have an acute onset of fever and cough or an acute onset of any three or more of the following signs or symptoms: fever, cough, general weakness/fatigue, headache, myalgia, sore throat, coryza, dyspnoea, and nausea/diarrhea/anorexia. We excluded individuals who had exclusively received non-inactivated vaccines. After obtaining written informed consent, we conducted a comprehensive questionnaire obtaining data on socio-demographic information, medical history, concomitant medications, and details of clinical presentation. Socioeconomic status (SES) was categorized based on monthly income, with three distinct tiers: low-income (less than USD 200 per month), middle-income (USD 200 to 800 per month), and high-income (more than USD 800 per month). High-risk occupation was defined by jobs or roles where workers were at an elevated risk of exposure to the virus due to the nature of their work environment, the tasks they performed, or the people they interacted with, e.g., healthcare, transportation, and food service workers.

All participants underwent nasopharyngeal swab collection for SARS-CoV-2 PCR testing (USTAR Biotechnologies, Hangzhou, China), with positives classified as cases and negatives as controls. Blood samples were collected for immunology analyses, and HIV testing was offered. We documented hospitalization outcomes where applicable. Vaccination status was verified through multiple methods, including physical inspection of vaccination cards, review of digital card images, and examination of clinic vaccination registers. Participants were classified as having a verified status if they either verbally confirmed no vaccination history or, for those reporting vaccination, provided proof through one of the aforementioned verification methods. Vaccination status was categorized as follows: partially vaccinated if the participant had received the first vaccine dose within 14 days prior to enrolment, fully vaccinated if more than 14 days had passed since the second vaccine dose, and boosted if the participant had received a third vaccine dose. Follow-up was conducted 2–8 weeks post-enrolment to assess symptom resolution.

### Statistical Analysis

We calculated the sample size using WHO-recommended methods for a test-negative case–control vaccine effectiveness study design [16]. Bivariate and multivariate logistic regression analyses were performed to investigate the relationships between various known COVID-19-associated demographic and health characteristics, vaccination status, and SARS-CoV-2 PCR positivity.

The primary outcome of interest was vaccine effectiveness at any time after receipt of at least one COVID-19 inactivated vaccine. In our secondary analyses, we estimated VE for each inactivated vaccine, boosting, time since vaccination, and enrolment location (clinic vs. hospital setting). These analyses were adjusted for covariates including age, sex, BMI, and various comorbidities such as diabetes, cardiovascular disease, HIV, tuberculosis, and cancers. Conditional logistic regression to assess the odds of testing SARS-CoV-2 PCR positive among the vaccinated vs. unvaccinated group was conducted.

We produced covariate-adjusted point estimates of vaccine effectiveness (VE), calculated as VE = (1 − aOR) × 100%. Conditional logistic regression models revealed the estimated vaccine effectiveness, expressed as odds ratios, along with corresponding confidence intervals. Our model adjusted for a comprehensive set of potential confounders including socio-demographic factors, lifestyle factors, comorbidities, and time since vaccination. Variable selection for the multivariate regression model involved identifying potential confounders based on the existing literature, biological plausibility, and statistical significance in univariate analyses. For each VE estimate, we calculated 95% confidence intervals and used 2-sided 5% significance to identify statistical differences between cases and controls. Matching variables by age and statistical analysis was performed in STATA (18th Edition, College Station, TX, USA).

This study was conducted with ethical approval from the Medical Research Council of Zimbabwe (MRCZ/A/2914).

## 3. Results

**Figure 1:**
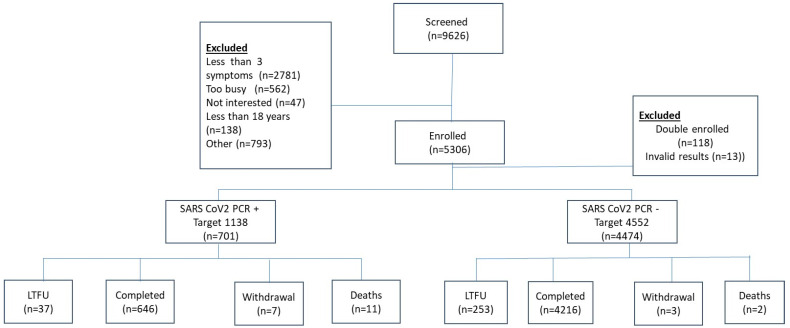
Consort diagram reflecting enrolment and outcomes for study participants.

We screened 9626 individuals for eligibility at 22 health facilities across Zimbabwe, ranging from primary healthcare centers providing outpatient clinical care to district and provincial hospitals, including national referral hospitals. The majority of the exclusions were due to participants having fewer than three symptoms (*n* = 2781) (Figure 1). A total of 5306 participants were enrolled. Following further exclusions for double enrolment (*n* = 118) and invalid laboratory results (*n* = 13), the final analytic cohort consisted of 5175 participants. Of these, 701 tested positive for SARS-CoV-2 by PCR (cases) and 4474 tested negative for SARS-CoV-2 by PCR (controls). Other reasons for screen failure included individuals who refused to participate, failure to meet the minimum age requirement for this study, i.e., <18 years, and non-eligible vaccine type (Supplementary Table S1).

Demographic data were available for all 5175 enrolled participants. There was a slight, non-significant difference in sex distribution between cases and controls, with 71.5% of cases being female compared to 67.9% of controls (*p* = 0.057) (Table 1). The median age was modestly higher among cases (37 years, IQR 27–48) compared to controls (36 years, IQR 26–44, *p* = 0.001). The distribution of age groups also showed a significant association with SARS-CoV-2 positivity, particularly in the 65+ age group (6.99% in cases vs. 2.55% in controls, *p* < 0.001). The ethnic distribution was predominantly Black African. Socioeconomic status did not significantly differ between cases and controls (*p* = 0.366). The majority of participants were enrolled at hospital-based outpatient clinics or emergency room settings (66.72%), with a significantly higher proportion of cases than controls among hospitalized inpatients (7.7% for cases vs. 0.38% for controls, *p* < 0.001). Community outpatient clinic enrolment was more common among controls than cases (33.57% vs. 21.26%). There was a significant difference in the highest level of education distribution, *p* = 0.004, between the cases and controls. A higher proportion of cases (8.4%) had only primary-level education compared to controls (5.0%). The distribution of PCR test results showed significant variation across provinces, with Harare accounting for 70.9% of the total sample and contributing the most positive tests, 78.3% (Table 1).

Cases had a slightly higher median BMI (26 (IQR 23–30) vs. 25 (IQR 22–29), *p* = 0.005). The burden of comorbid conditions differed significantly between cases and controls (Table 1). Cases had a higher proportion of participants with hypertension (19.16% vs. 14.15%, *p* = 0.001), asthma (3.86% vs. 2.51%, *p* = 0.039), and tuberculosis (1.66% vs. 0.78%, *p* = 0.034) compared to controls. HIV prevalence was high in both groups but not significantly different (22.07% of cases vs. 22.75% of controls, *p* = 0.698). Notably, a significantly higher proportion of cases than controls were pregnant women (16.19% vs. 8.68%, *p* < 0.001) (Table 2). The most common comorbid conditions across the entire study population were HIV (22.66%), hypertension (14.83%), and obesity (BMI ≥ 30) (24.83%) (Table 1). Among the HIV-infected, 83.4% were on ART (Table 2).

Common symptoms like acute fever (50.4% vs. 38%, *p* < 0.001), muscle pain (37.7% vs. 33.5%, *p* = 0.031), general weakness (65.9% vs. 57.9%, *p* < 0.001), and altered mental state (4.6% vs. 3.1%, *p* = 0.042) were significantly more prevalent in cases compared to controls, and prior COVID-19 infection was also more common among cases (36.8% vs. 32.6%, *p* = 0.030) (Supplementary Table S2).

Overall, the majority of the population, 82.9%, reported to have been vaccinated, and only 877 (17.1%) of them had not been vaccinated. Among those reporting their vaccination status, 42.1% of participants were fully vaccinated (Table 3) and 33.4% had received booster doses. Partial vaccination was observed in 7.2% of the cohort. Younger individuals (18–49) had higher rates of partial and full vaccination, while older individuals (50+) had higher rates of booster vaccination (participants aged 50–64 (49.9%) and 65+ (51.9%)) (Table 4). Sinopharm was the most commonly utilized vaccine; 57.4% of the cohort had been vaccinated with BBIBP-CorV, 39.5% with Coronovac, and 0.4% with BBV152 Covaxin (Table 4). Age-wise distribution showed a similar pattern, with BBIBP-CorV being the dominant vaccine across all age groups.

The median time since the last vaccine dose for the overall vaccinated group was 434 days (IQR: 266–616) (Table 3). The median time since the last booster dose was 310 days (IQR: 205–409 days). This varied across age groups, with the longest duration observed in the 65+ group (median 368 days (IQR: 307–467 days)) (Table 3).

There were significant differences in median age, age group distribution, socio-economic status, and enrolment site between vaccinated and unvaccinated individuals (Supplementary Table S3). Sex (*p* = 0.937) and ethnicity (*p* = 0.773) distributions were similar across both groups, with no significant differences noted. There were significantly fewer individuals with tuberculosis among the vaccinated compared to the unvaccinated individuals (0.7% vs. 1.9%, *p* = 0.002) (Supplementary Table S4). We successfully verified the vaccination status of 3297/4292 (76.8%) of those reporting vaccination, primarily through vaccine card inspection and digital image submission of vaccination cards (Supplementary Table S5).

In the analysis of participants with verified vaccination status within the study cohort, both bivariate and multivariate logistic regression models were used to assess the association between demographic variables, health indicators, and vaccination status (Table 5). In the multivariate analysis, significant risk factors included high BMI (aOR = 1.44, 95% CI: 1.127–1.835), hospital enrolment (aOR = 1.49, 95% CI: 1.231–1.791), previous COVID diagnosis (aOR = 2.85, 95% CI: 2.051–3.952), hypertension (aOR = 1.64, 95% CI: 1.225–2.206), and working in a high-risk occupation (aOR = 2.98, 95% CI: 2.430–3.643). Protective factors included low BMI (aOR = 0.50, 95% CI: 0.312–0.814) and active tuberculosis (aOR = 0.44, 95% CI: 0.211–0.904) (Table 5).

**Figure 2:**
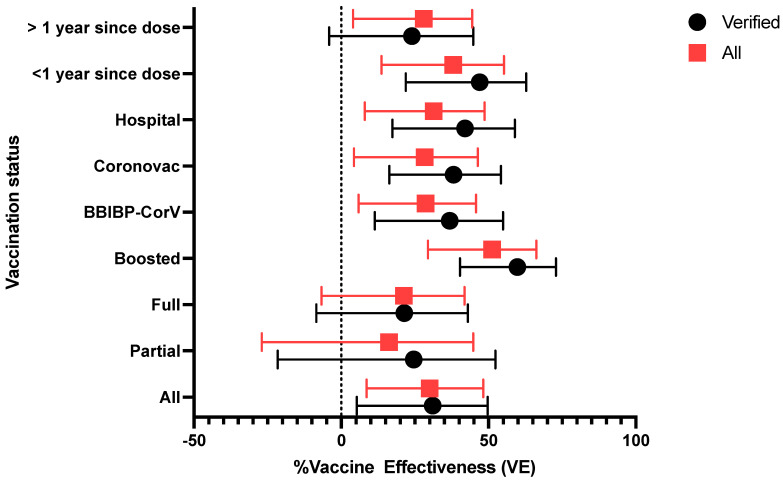
Adjusted vaccine effectiveness (VE) by vaccination status and subgroup. Adjusted VE percentages with 95% confidence intervals for various vaccination statuses and subgroups. Black circles represent verified participants; red squares represent all participants. The *x*-axis shows VE, ranging from −50% to 100%, with error bars indicating 95% confidence intervals.

The adjusted vaccine efficacy (VE) against SARS-CoV-2 infection was evaluated across various subgroups within the study cohort (Table 6). Overall, the adjusted VE was 31% (95% CI: 5.3% to 49.7%) among verified participants and 30% (95% CI: 8.6% to 48.2%) among all participants. Participants enrolled from hospitals showed a higher VE of 42% (95% CI: 17.4% to 58.9%) among verified participants. VE was similar in those who received the BBIBP-CorV and Coronovac vaccines, with adjusted VEs of 36.8% (95% CI: 11.4% to 54.9%) and 38.1% (95% CI: 16.3% to 54.2%), respectively, among verified participants (Figure 2).

Individuals who had received booster doses demonstrated the highest vaccine effectiveness (VE) at 59.8% (95% CI: 40.3% to 72.9%) in the verified group; this compared to 21.4% (95% CI: −8.5% to 43%) for fully vaccinated individuals and 24.6% (95% CI: −21.6% to 52.3%) for partially vaccinated participants. The time since vaccination appeared to impact effectiveness. The median time post-vaccination was 434 days (IQR: 266 to 616 days) (Table 4). Individuals who received their last vaccine dose within the past year demonstrated a higher VE of 47% (95% CI: 21.9% to 62.8%) compared to those vaccinated more than a year ago, where VE was no longer evident at 24% (95% CI: −4.1% to 44.8%).

A sensitivity analysis was conducted to compare adjusted vaccine effectiveness (VE) by vaccination status and various subgroups, focusing on all participants and those whose vaccination status was successfully verified through a rigorous process. The vaccination verification involved checking vaccination cards at enrolment or follow-up, conducting phone calls to obtain vaccination details and digital pictures of the cards, making home visits, and verifying records at the clinics. Despite these efforts, only 79.2% of participants who reported being vaccinated were successfully verified (Table 3). In the analysis, verified participants consistently demonstrated higher VE across all subgroups compared to the broader group that included those who reported vaccination but could not be verified. For instance, boosted individuals exhibited the highest VE, with 59.8% (95% CI: 40.3% to 72.9%) in the verified group, compared to lower VE (51.2% (95% CI: 29.4% to 66.2%)) for all those who reported vaccination. Similarly, the effectiveness of the Coronovac and BBIBP-CorV vaccines was higher among verified participants (Figure 2).

## 4. Discussion

This study provides valuable insights into the real-world effectiveness of inactivated COVID-19 vaccines in Zimbabwe, revealing a modest level of protection against symptomatic SARS-CoV-2 infection, with notable variations across different vaccination statuses and subgroups.

The overall vaccine effectiveness (VE) of 31% (95% CI: 5.3–49.7%) for the prevention of symptomatic disease among verified vaccinated individuals was lower than the efficacy reported in initial clinical trials [5,6,7,8]. A VE study in Morocco that was conducted prior to the emergence of the Omicron variant observed a higher VE of 67% (95% CI: 43% to 80%) [10]. Our data, however, are more closely aligned with a large VE study conducted in Shanghai, China, that enrolled participants before and after the emergence of the Omicron variant. This study demonstrated a VE of 16.3% (95% CI: 15.4% to 17.2%) against symptomatic disease, increasing to 88.6% (95% CI: 85.8% to 90.9%) for severe disease and 91.7% (95% CI: 86.95% to 94.5%) for death [19]. In our study, the relatively low VE for symptomatic disease is likely attributable to the predominance of Omicron variants during our study period. The omicron variant and its subvariants have demonstrated an increased ability to evade vaccine-induced immunity [17,20]. The VE that we observed was slightly higher for symptomatic disease with Omicron variants than that observed in the Shanghai, China, cohort and a recent metanalysis (VE 16.4% (95% CI: 9.5 to 22.8% for omicron)) [21]. We hypothesize that this may reflect hybrid immunity induced by both vaccination and recent infection, given the high levels of circulating virus [22,23].

We observed a higher VE of 42% (95% CI: 17.4–58.9%) among hospital-enrolled participants. This suggests stronger protection against more severe forms of COVID-19 requiring hospital care, consistent with findings from other countries [11,12,19]. However, the low proportion of severely ill participants in our study (1.3% hospitalized) limited our ability to evaluate VE against severe disease directly. We conducted our study at a period when Omicron was dominant and had been associated with less severe disease on the continent [24,25].

The effectiveness of booster doses is a significant finding, with VE increasing to 59.8% (95% CI: 40.3–72.9%) for boosted individuals. This underscores the importance of booster vaccination programs, especially in the context of emerging variants, where homologous boosting with inactivated vaccines against the original variants has been shown to confer protection against emergent Delta and Omicron variants [26]. The relative VE of 27.4% (95% CI: 8.8–42.8%) for boosted compared to fully vaccinated individuals further reflects the additional protection provided by booster doses.

Time since vaccination emerged as a critical factor influencing effectiveness. VE dropped significantly to 24% (95% CI: −4.1% to 44.8%) at ≥1 year post-vaccination, compared to 47% (95% CI: 21.9% to 62.8%) for those vaccinated within the past year. This waning effectiveness aligns with observations from other studies and vaccine platforms [27,28]. The low protection could also be explained by the extended time since the participants’ last dose of vaccination, with a median time of 434 days. This extended interval likely contributed to the reduced protection, which could have been due to waning immunity, variation in immune response, or evolution of the virus, as observed in other studies [29,30].

We observed similar VE for BBIBP-CorV (36.8%, 95% CI: 11.4–54.9%) and Coronovac (38.1%, 95% CI: 16.3–54.2%) vaccines, indicating the comparable performance of these inactivated vaccines in the study population.

The vaccination rate in our study population (83%) was notably higher than the national average. In Zimbabwe, the vaccine rollout initially targeted older age groups but was expanded to everyone above 12 years by August 2021. As of 31 December 2023, national vaccination coverage stood at 51% for the first dose, 38% for the second dose, and 15% for the booster shot (WHO COVID-19 Dashboard, Zimbabwe, Zimbabwe). This discrepancy likely reflects a combination of factors, including potential selection bias towards individuals more likely to seek healthcare and the higher socioeconomic status (SES) of our study population. Indeed, 46.3% of our participants fell into the middle or high SES categories, which is substantially higher than the national average, where about 80% of the urban population has a monthly income below USD 200 [31].

This study employed meticulous vaccination verification methods, achieving a verification rate of 79.2% among those reporting vaccination. Vaccination verification is challenging in many settings including Africa where records are not digitized, and cards may be easily lost. In addition, with COVID, there were significant challenges due to card falsification. The verification rate that we achieved was relatively high [32] due to the intense resources dedicated to following up on all study participants and documenting vaccination. However, despite this, we acknowledge the potential for misclassification bias, particularly among those reporting no vaccination, which we accepted at face value. We addressed this bias through a sensitivity analysis for VE by separating the analysis of VE for those whose status was unverified or who self-reported vaccination.

The observed differences between cases and controls likely arose by chance because both groups were drawn from the same sample population presenting at health centers. Since the individuals in both groups were selected from the same setting and under similar circumstances, any differences were not necessarily due to any underlying systematic differences but rather due to the nature of the disease, age effects, comorbidity effects, and crowding and social factors, some of which were not fully explored in this analysis. The differences in age were adjusted for in the analysis by matching the cases and controls. These results highlight areas for further research, such as investigating the biological and social drivers and the relevance of these differences in this setting.

Several limitations should be considered when interpreting our results. The test-negative design, while practical for real-world effectiveness studies, is subject to potential biases, including differences in misclassification bias and the impact of healthcare-seeking behavior in the analyzed cohort. Additionally, we did not analyze the vaccine effectiveness of the inactivated vaccines in individuals who experienced breakthrough infection as this was driven by recall rather than documented prior COVID-19 testing, which was difficult to obtain in a real-world setting. The study cohort, predominantly young and female, may limit generalizability but reflects common health system utilization trends in Africa [33]. Due to the uneven sample sizes, differences in exposure risks, lack of statistical power in smaller provinces, and confounding factors, comparing VE by province using this dataset would lead to unreliable and potentially misleading conclusions.

## 5. Conclusions

The study findings provide evidence of reduced effectiveness of inactivated COVID-19 vaccines against symptomatic Omicron infection in Zimbabwe, consistent with global trends. The increased effectiveness with boosters and the significant waning of protection over time underscore the need to consider potential annual boosting strategies. However, future boosters may require optimized vaccines that better match circulating strains. In resource-limited settings, special consideration must be given to the cost-effectiveness of vaccination programs, given the relatively low disease severity observed. This may necessitate a targeted approach, focusing on vaccinating the most high-risk groups. Larger studies will be required to define these high-risk populations in Africa that would benefit most from continued vaccination efforts.

We anticipate that the results from this study will inform public health decision-making in Zimbabwe and other sub-Saharan countries with similar demographic and epidemiological profiles. As the global community continues to navigate the challenges posed by COVID-19, real-world effectiveness data from diverse settings remain significant for shaping equitable, cost-effective, and tailored pandemic responses. Future strategies should prioritize the rapid deployment of effectiveness studies in target populations to better inform regional epidemic responses.

## Figures and Tables

**Table 1:** Sociodemographic characteristics and medical history of study population.

Variable	Controls	Cases	Total	*p*-Value
**Age (median, IQR)**	36 (26–44)	37 (27–48)	36 (27–45)	0.001
**Age group**	***n*** ** = 4474**	***n*** ** = 701**	***n*** ** = 5175**	
18–49	3805 (85.05)	547 (78.03)	4352 (84.10)	<0.001
50–64	555 (12.41)	105 (14.98)	660 (12.75)	
65+	114 (2.55)	49 (6.99)	163 (3.15)	
**Sex**	***n*** ** = 4474**	***n*** ** = 701**	***n*** ** = 5175**	
Male	1437 (32.12)	200 (28.53)	1637 (31.63)	0.057
Female	3037 (67.88)	501 (71.47)	3538 (68.37)	
**Enrolment site**	***n*** ** = 4474**	***n*** ** = 701**	***n*** ** = 5175**	
Hospital outpatient or ER	2955 (66.05)	498 (71.04)	3453 (66.72)	<0.001
Hospitalized In-patient	17 (0.38)	54 (7.70)	71 (1.37)	
Community Outpatient	1502 (33.57)	149 (21.26)	1651 (31.90)	
**Socio-economic status**	***n*** ** = 4458**	***n*** ** = 700**	***n*** ** = 5158**	
Low	2378 (53.34)	391 (55.86)	2769 (53.68)	0.366
Middle	1972 (44.24)	290 (41.43)	2262 (43.85)	
High	108 (2.42)	19 (2.71)	127 (2.46)	
**Ethnicity**	***n*** ** = 4463**	***n*** ** = 699**	***n*** ** = 5162**	
Asian, not Indian	7 (0.16)	1 (0.14)	8 (0.15)	0.007
Black	4444 (99.57)	689 (98.57)	5133 (99.44)	
Indian	2 (0.04)	2 (0.29)	4 (0.08)	
Mixed	3 (0.07)	3 (0.43)	6 (0.12)	
White	5 (0.11)	3 (0.43)	8 (0.15)	
Other	2 (0.04)	1 (0.14)	3 (0.06)	
**Highest level of education**	***n*** ** = 4474**	***n*** ** = 701**	***n*** ** = 5175**	
Not attended school	17 (0.4)	5 (0.7)	22 (0.4)	0.004
Primary	223 (5.0)	59 (8.4)	282 (5.5)	
Secondary	2861 (64.0)	426 (60.8)	3287 (63.5)	
Tertiary	959 (21.4)	141 (20.1)	1100 (21.3)	
Degree	390 (8.7)	64 (9.1)	454 (8.8)	
Missing	24 (0.5)	6 (0.9)	30 (0.6)	
**Province**	***n*** ** = 4474**	***n*** ** = 701**	***n*** ** = 5175**	
Harare	3120 (69.7)	549 (78.3)	3669 (70.9)	<0.001
Manicaland	215 (4.8)	7 (1.0)	222 (4.3)	
Mashonaland Central	152 (3.4)	3 (0.4)	155 (3.0)	
Mashonaland East	465 (10.4)	70 (10.0)	535 (10.3)	
Mashonaland West	372 (8.3)	46 (6.6)	418 (8.1)	
Masvingo	150 (3.4)	26 (3.7)	176 (3.4)	

**Table 2:** Comorbid conditions in cases and controls.

Variable	Controls (%)	Cases (%)	Total (%)	*p*-Value
**BMI (median, IQR)**	25 (22–29)	26 (23–30)	25 (22–30)	0.005
**BMI range**	***n*** ** = 4474**	***n*** ** = 701**	***n*** ** = 5175**	
<18	103 (2.30)	21 (3.00)	124 (2.40)	0.013
18–24.9	1899 (42.45)	249 (35.52)	2148 (41.51)	
25–29.9	1285 (28.72)	226 (32.24)	1511 (29.20)	
≥30	1094 (24.45)	191 (27.25)	1285 (24.83)	
Missing	93 (2.08)	14 (2.00)	107 (2.07)	
**Medical comorbidities**				
Hypertension	631/4458 (14.15)	133/694 (19.16)	764/5152 (14.83)	0.001
Diabetes	128/4457 (2.87)	26/694 (3.75)	154/5151 (2.99)	0.208
Dementia	5/4457 (0.11)	1/694 (0.14)	6/5151 (0.12)	0.819
Chronic kidney disease	10/4457 (0.22)	3/694 (0.43)	13/5151 (0.25)	0.309
Asthma	112/4467 (2.51)	27/700 (3.86)	139/5167 (2.69)	0.039
Tuberculosis	29/3719 (0.78)	10/602 (1.66)	39/4321 (0.9)	0.034
HIV	992/4361 (22.75)	149/675 (22.07)	1141/5036 (22.66)	0.698
On ART	831/992 (83.8)	121/149 (81.2)	952/1141 (83.4)	0.424
Currently smoking	287/4424 (6.49)	25/693 (3.61)	312/5117 (6.1)	0.003
Cancer	20/4458 (0.45)	7/694 (1.01)	27/5152 (0.52)	0.057
Pregnancy	218/2511 (8.68)	68/420 (16.19)	286/2931 (9.76)	<0.001
High-risk occupation	1986/4462 (44.51)	251/701(35.81)	2237/5163 (43.33)	<0.001
Previous COVID diagnosis	1436/4408 (32.58)	254/691 (36.76)	1690/5099 (33.14)	0.03

**Table 3:** Vaccination status among cases and controls.

Variable	Controls	Cases	Total	*p*-Value
Vaccinated	3720/4474 (83.1)	572/701 (81.6)	4292/5175 (82.9)	0.276
Verified	2879/3618 (79.6)	418/544 (76.8)	3297/4162 (79.2)	0.143
**Vaccine Brand**	***n*** ** = 3720**	***n*** ** = 572**	***n*** ** = 4292**	
BBIBP-CorV (Sinopharm)	2128 (57.2)	337 (58.9)	2465 (57.4)	0.601
Coronovac (Sinovac)	1483 (39.9)	212 (37.1)	1695 (39.5)	
BBV152 (Covaxin)	16 (0.4)	3 (0.5)	19 (0.4)	
Other *	16 (0.4)	2 (0.3)	18 (0.4)	
Unknown/not sure **	77 (1.8)	18 (2.8)	95 (2.2)	
Vaccination status ***	***n*** ** = 4372**	***n*** ** = 675**	***n*** ** = 5047**	
Unvaccinated	748 (17.1)	129 (19.1)	877(17.4)	0.057
Partial vaccination	309 (7.1)	55 (8.2)	364 (7.2)	
Fully vaccinated	1827 (41.8)	296 (43.9)	2123 (42.1)	
Boosted	1488 (34.0)	195 (28.9)	1683 (33.4)	

* Sputnik V (Gamaleya, Moscow, Russia) and Janssen Pharmaceuticals Lieden, The Netherlands). ** Participants were not sure of the vaccine names they received. *** Participants with a known number of doses received.

**Table 4:** Vaccination status and median time since last vaccine for study participants by age group with known vaccination status.

	Total (%)	Age (18–49 Y)	Age (50–64 Y)	Age ≥ 65 Years
Vaccination status	*n* = 5047	*n* = 4244	*n* = 645	*n* = 158
Partial vaccination	364 (7.2)	338 (8.0)	23 (3.6)	3 (1.9)
Full vaccination	2123 (42.1)	1827 (43.1)	246 (38.1)	50 (31.7)
Booster vaccination	1683 (33.4)	1279 (30.1)	322 (49.9)	82 (51.9)
**List of vaccine brands**	*n* = 4292	*n* = 3547	*n* = 605	*n* = 140
BBIBP-CorV (Sinopharm)	2465 (57.4)	2054 (57.9)	338 (55.9)	73 (52.1)
Coronovac (Sinovac)	1695 (39.5)	1382 (39.0)	251 (41.5)	62 (44.7)
BBV152 (Covaxin)	19 (0.4)	15 (0.4)	4 (0.7)	0 (0.0)
Other *	18 (0.4%)	16 (0.5)	2 (0.3)	0
Unknown/not sure	95 (2.2%)	80 (2.3)	10 (1.7)	5 (3.6)
**Time since vaccination**				
Days since last vaccine dose (median, IQR)	434 (266–616)	433 (260–620.5)	440 (294–602)	403 (312–543)
Days since booster vaccine dose (median, IQR)	310(205–409)	300 (193–399)	335 (244–438)	368 (307–467)

* means Gam-COVID-Vac (Sputnik V,) Gamaleya Research Institute of Epidemiology and Microbiology, Moscow, Russian, and means Jcovden (Jansen) Janssen Vaccines, Leiden, Netherlands.

**Table 5:** Odds ratios and confidence intervals from bivariate and multivariate analyses of confirmed vaccination status within the study cohort by demographic variables and health indicators.

	Total ^1^	Univariate Analysis			Multivariate Analysis		
Variable		OR	95% CI	*p*-Value	aOR	95% CI	*p*-Value
Sex (ref female)	1630	0.99	0.850–1.162	0.937			
BMI (ref 18–24.9)							
<18	124	0.61	0.409–0.907	0.015	0.50	0.312–0.814	0.006
25–29.9	1506	1.31	1.101–1.552	0.002	1.19	0.965–1.455	0.112
≥30	1281	2.00	1.634–2.443	<0.001	1.44	1.127–1.835	0.004
Clinic type (ref outpatient)							
Hospital	3448	2.42	2.088–2.817	<0.001	1.49	1.231–1.791	<0.001
In-patient	71	0.78	0.463–1.297	0.333	0.58	0.316–1.051	0.065
Previous COVID diagnosis	1684	1.49	1.265–1.756	<0.001	2.85	2.051–3.952	<0.001
HIV-positive	1136	0.89	0.745–1.049	0.159			
Active TB	39	0.37	0.187–0.717	0.003	0.44	0.211–0.904	0.026
Asthma	139	1.59	0.942–2.699	0.082			
Hypertension	762	1.96	1.532–2.509	<0.001	1.64	1.225–2.206	0.001
Diabetes mellitus	153	2.08	1.195–3.623	0.010			
PCR-positive	699	0.89	0.725–1.097	0.280			
High-risk occupation	2234	3.83	3.200–4.574	<0.001	2.98	2.430–3.643	<0.001
Age group							
50–64 years	658	2.53	1.895–3.377	<0.001			
65+ years	163	1.38	0.881–2.158	0.159			

^1^ Total represents that total number with reference indication included in the model.

**Table 6:** Adjusted subgroup VE.

	Verified Participants			All Participants		
Subgroup	Total (*n*)	Case (*n*, %)	Adjusted VE, % (95% CI)	Total (*n*)	Case (*n*, %)	Adjusted VE, % (95% CI)
Overall	4184	547 (13.1)	31 (5.3%, 49.7%)	5175	701 (13.5)	30 (8.6%, 48.2%)
Hospital-enrolled	2848	429 (15.1)	42 (17.4%, 58.9%)	3524	552 (15.7)	31.3 (8.0%, 48.7%)
**Vaccine Brand**						
Sinopharm	1877	240 (12.8)	36.8 (11.4%, 54.9%)	2469	338 (13.7)	28.6 (5.9%, 45.8%)
Sinovac	1392	175 (12.6)	38.1 (16.3%, 54.2%)	1702	213 (12.5)	28.3 (4.3%, 46.3%)
**Vaccination status**						
Partially	220	28 (12.7)	24.6 (−21.6%, 52.3%)	364	55 (15.1)	16.2 (−27%, 44.8%)
Fully	1627	225 (13.8)	21.4 (−8.5%, 43%)	2123	296 (13.9)	21.2 (−6.7%, 41.8%)
Boosted	1446	165 (11.4)	59.8 (40.3%, 72.9%)	1683	195 (11.6)	51.2 (29.4%, 66.2%)
**Time since vaccination**						
<1 year	1346	148 (11.0)	47 (21.9%, 62.8%)	1718	208 (12.1)	38 (13.7%, 55.2%)
>1 year	1947	270 (13.9)	24 (−4.1%, 44.8%)	2452	338 (13.8)	28 (4%, 44.4%)

## Data Availability

Currently we do not have publicly archived datasets but can make the data available upon request.

## Supplementary Materials

The following supporting information can be downloaded at https://www.mdpi.com/article/10.3390/vaccines12121303/s1: Table S1. Reasons for Screening Failure. Table S2. Symptoms on presentation. Table S3. Vaccination status and demographic profile. Table S4. Comorbid conditions by vaccination status. Table S5. Table of vaccine verification.
