# Supplementary material for: The Real-World Effectiveness of Inactivated COVID-19 Vaccines in Zimbabwe During the Omicron Variant Dominance: A Test-Negative Case–Control Study
Source: Vaccines (Basel). 2024 Nov 22;12(12):1303. doi: 10.3390/vaccines12121303 (PMC11680042; doi:10.3390/vaccines12121303)
Supplement: Supplementary file 1 [file vaccines-12-01303-s001.zip › vaccines-3247424-supplementary.pdf]

Supplementary Tables

Table S1. Reasons for Screening Failure

Reason	N
Fewer than 3 symptoms	2781
Under 18 years of age	138
Not willing to participate	355
Non eligible vaccine type	393
Other	
Not Interested	438
Fear of blood draw and swab draw	20
Feeling too sick	136
Fear of COVID-19 NP[1] swab	38
RA discretion (e.g. lives far away, intoxicated)	21
Total Screened	9626
Total Screen Failure	4320 (44.9%)

Table S2. Symptoms on presentation

Variable	Total	Cases (%)	Controls (%)	p-value
**Symptoms**				
Acute Fever	2052/5168 (39.7)	353/701 (50.4)	1699/4467 (38.0)	<0.001
Acute Cough	4063/5168 (78.6)	577/701 (82.3)	3486/4467 (78.0)	0.010
General weakness	3049/5168 (59.0)	462/701 (65.9)	2587/4467 (57.9)	<0.001
Headache	4381/5168 (84.8)	565/701 (80.6)	3816/4467 (85.4)	0.001
Muscle pains	1761/5168 (34.1)	264/701 (37.7)	1497/4467 (33.5)	0.031
Sore throat	3472/5168 (67.2)	453/701 (64.6)	3019/4467 (67.6)	0.120
Runny nose	3539/5168 (68.5)	483/701 (68.9)	3056/4467 (68.4)	0.796
Shortness of breath	1390/5168 (26.9)	190/701 (27.1)	1200/4467 (26.9)	0.894
Loss of appetite	2649/5168 (51.3)	342/701 (48.8)	2307/4467 (51.7)	0.159
Diarrhea	489/5168 (9.5)	58/701 (8.3)	431/4467 (9.7)	0.248
Altered mental status	170/5168 (3.3)	32/701 (4.6)	138/4467 (3.1)	0.042

[1] NP: Nasopharyngeal

Table S3. Vaccination status and demographic profile

Variable	Vaccinated (%)*	Unvaccinated (%)	Total (%)	p-value
**Age (median, IQR)**	37 (27 - 46)	30 (23 - 39)	36 (27 - 45)	<0.001
**Age-group**				
18-49	3531/4274 (82.6)	800/877 (91.2)	4347/5151 (84.1)	<0.001
50-64	603/4274 (14.1)	54/877 (6.2)	659/5151 (12.8)	
65+	140/4274 (3.3)	23/877 (2.6)	163/5151 (3.2)	
**Gender**				
Male	2925/4274 (68.4)	599/877 (68.3)	3535/5151 (68.4)	0.937
Female	1349/4274 (31.6)	278/877 (31.7)	1634/5151 (31.6)	
**Enrolment site**				
Hospital outpatient or ER	1215/4274 (28.4)	423/877 (48.2)	1638/5151 (31.8)	<0.001
Hospitalized In-patient	3010/4274 (70.4)	432/877 (49.3)	3442/5151 (66.8)	
Community Outpatient	49/4274 (1.2)	22/877 (2.5)	71/5151 (1.4)	
**Socio-economic status**				
Low	2145/4282 (50.3)	611/876 (69.8)	2756/5140 (53.6)	<0.001
Middle	2010/4282 (47.1)	247/876 (28.2)	2257/5140 (43.9)	
High	109/4264 (2.6)	18/876 (2.1)	127/5140 (2.5)	
**Ethnicity**				
Asian, not Indian	7/4266 (0.2)	1/877 (0.1)	8/5143 (0.2)	0.773
Black	4243/4266 (99.5)	871/877 (99.3)	5114/5143 (99.4)	
Indian	3/4266 (0.1)	1/877 (0.1)	4/5143 (0.1)	
Mixed	5/4266 (0.1)	1/877 (0.1)	6/5143 (0.1)	
White	5/4266 (0.1)	3/877 (0.3)	8/5143 (0.2)	
Other	3/4266 (0.1)	0	3/5143 (0.1)	

Table S4. Comorbid conditions by vaccination status

Variable	Vaccinated N(%)	Unvaccinated N(%)	Total N(%)	p-value
**BMI (median, IQR)**	25 (22 – 30)	24 (21 – 28)	25 (22 – 30)	<0.001
**BMI range**				
<18	87/4177 (2.1)	37/873 (4.2)	124/5050 (2.5)	<0.001
18-24.9	1699/4177 (40.7)	440/873 (50.4)	2139/5050 (42.4)	
25-29.9	1257/4177 (30.1)	249/873 (28.5)	1506/5050 (29.8)	
≥30	1134/4177 (27.2)	147/873 (16.8)	1281/5050 (25.4)	
**Medical comorbidities**				
Hypertension	684/4257 (16.1)	78/877 (8.9)	762/5134 (14.8)	<0.001
Diabetes	139/4256 (3.3)	14/877 (1.6)	153/5133 (3.0)	0.008
Dementia	4/4256 (0.1)	2/877 (0.2)	6/5133 (0.1)	0.465

Chronic kidney disease	10/4256 (0.2)	3/877 (0.3)	13/51533 (0.3)	0.566
Asthma	123/4272 (2.9)	16/877 (1.8)	139/5149 (2.7)	0.079
Tuberculosis	26/3633 (0.7)	13/674 (1.9)	39/4307 (0.9)	0.002
HIV status	923/4147 (22.3)	213/871 (24.5)	1136/5018 (22.6)	0.159
On ART	779/794 (98.1)	170/182 (93.4)	949/976 (97.2)	<0.001
Currently smoking	229/4223 (5.4)	81/876 (9.3)	310/5099 (6.1)	<0.001
Cancer	26/4257 (0.6)	1/877 (0.1)	27/5134 (0.5)	0.064
Pregnancy	194/2464 (7.9)	92/457 (20.1)	286/2921 (9.8)	<0.001
High risk occupation	2062/4268 (48.3)	172/877 (19.6)	2234/5145 (43.3)	<0.001
Previous COVID diagnosis	1456/4210 (34.6)	228/871 (26.2)	1684/5081 (33.1)	<0.001

Table S5. Table of vaccine verification

**Source of vaccination information**	**N (%)**
Confirmed by phone call later	274 (8.3)
Digital image submission	372 (11.3)
Inspection of Clinic vaccination register	41 (1.2)
Vaccine card inspection	2610 (79.2)
